# Short-term data suggests cognitive benefits in the elderly with single-implant overdentures

**DOI:** 10.1038/s41432-024-00999-4

**Published:** 2024-04-03

**Authors:** Kelvin I. Afrashtehfar, Carlos A. Jurado, Salem H. Abu Fanas, Massimo Del Fabbro

**Affiliations:** 1 Department of Reconstructive Dentistry and Gerodontology, School of Dental Medicine, University of Bern, Bern, Switzerland; 2 College of Dentistry, Ajman University, Ajman City, UAE; 3 Dental Consultant, Private Practice limited to Prosthodontics & Implantology, Abu Dhabi, UAE; 4 Director of the Operative Dentistry Division, Department of General Dentistry, College of Dentistry, University of Tennessee Health Science Center, Memphis, TN USA; 5 Dean of the College of Dentistry, Ajman University, Ajman City, UAE; 6 Department of Biomedical, Surgical and Dental Sciences, Università degli Studi di Milano, Milan, Italy; 7 UOC Maxillofacial Surgery and Dentistry, Fondazione IRCCS Ca’ Granda, Ospedale Maggiore Policlinico, Milan, Italy

**Keywords:** Dental implants, Gerodontics

## Abstract

**Design:**

This study was an extension of a randomized crossover clinical trial approved by the institutional ethics committee (approval number: D2014–148) and adhered to the CONSORT guidelines. The original study juxtaposed patient contentment with single-implant overdentures (1-IODs) against conventional complete dentures (CCDs), with patient satisfaction being the primary focus. In this follow-up study, the cognitive function of edentulous patients receiving 1-IODs was assessed, specifically monitoring for the emergence of mild cognitive impairment (MCI) throughout a three-year period.

Patient outcomes were systematically recorded at predetermined intervals: initially, two months post-1-IOD placement, after one year (with groups alternated between denture types at eight-month marks), then after two and three years. A prosthodontist with a decade of expertise performed all denture-related procedures. This follow-up emphasized the cognitive outcomes using the Montreal Cognitive Assessment (MoCA-J), considering it alongside previously documented results on masticatory function, bone resorption, survival rates, and patient-reported outcomes.

**Case selection:**

Between 2015 and 2016, a follow-up study enrolled edentulous patients over 50 years of age who were proficient in Japanese, had sufficient mandibular bone for implants, and were free of systemic health issues and habits that could impact oral health. The participants were randomly divided into two groups after receiving a central mandibular implant. Group 1 initially used 1-IODs, and Group 2 used unloaded CCDs. After two months and subsequent periods, they swapped denture types. Eventually, all patients chose 1-IODs for continued use. Implant success was monitored over three years.

The design featured block randomization and accounted for a sample size of 22, determined to be sufficient for evaluating the primary outcome of patient satisfaction. All patients underwent careful allocation and received customized dental interventions, with detailed radiographic planning and surgical precision guiding the implantation process.

**Data analysis:**

Multivariable linear mixed models were used to assess within-group changes in both overall and specific cognitive function scores across five timepoints. Age, assessment interval, and upper jaw denture status were incorporated as consistent variables, while individual participants were considered variable elements in the analysis. SPSS software version 22.0 was utilized to conduct the statistical tests, and a *p* value threshold of 0.05 was predetermined to establish statistical significance.

**Results:**

Twenty-two patients with edentulous mandibles received 1-IODs. Memory and executive functions saw significant score increases at multiple timepoints over the three-year period, with statistical significance. Though one participant dropped out and another passed away, and two did not complete the 3-year follow-up, the remaining 18 participants provided comprehensive data. Age and type of maxillary denture were significant factors, influencing MoCA-J scores with older participants and those with fixed dentures showing lower scores in certain domains. Overall, the findings illustrated the positive correlation between 1-IODs and cognitive function in older adults.

**Conclusions:**

Older adults with no natural teeth left in their mandible showed improved cognitive function after one and three years of using 1-IODs, as reflected by their total and specific cognitive domain scores. The study suggests that such implant therapy may offer protective benefits against cognitive decline, demonstrating clinical relevance for patient care, regardless of the maxillary arch (antagonist) condition.

## A Commentary on

**Komagamine Y, Kanazawa M, Miyayasu A** **et al**.

The effect of single-implant overdentures on cognitive function in older adults: A 3-year follow-up report. *J Dent* 2023; **136**:104632.

**GRADE Rating**: 

## Commentary

Dental implants are commonly utilized for their ability to restore both the functionality and appearance of missing teeth^1,2^. Early detection and intervention are crucial for mild cognitive impairment (MCI), an early indicator of dementia and a significant concern for elderly care^3^. Edentulousness, or tooth loss, can disrupt brain function by affecting hippocampal connectivity and the trigeminal nerve, increasing the risk of cognitive decline^4^. However, well-fitting dentures, especially two implant-assisted overdentures (2-IODs), are associated with higher cognitive function^5^. Despite this, a study indicates that edentulous patients with dentures still face a greater risk of MCI, suggesting the need for ongoing research into the cognitive benefits of implant overdentures (IODs)^6^. The Montreal Cognitive Assessment (MoCA) is more effective than the Mini-Mental State Examination (MMSE) for detecting mild cognitive impairment (MCI)^7^, with studies indicating its heightened sensitivity in patients with diabetes and heart failure^8^. The randomized controlled trial (RCT) by Komagamine et al. hypothesized improved cognitive performance on the MoCA-J for those with single implant overdentures (1-IODs) compared to conventional denture wearers.

The RCT has several strengths. Firstly, the study design adheres to CONSORT guidelines and it addressed a clear, focused research question concerning the cognitive effects of 1-IODs on edentulous patients. Randomization and blinding procedures were properly described, which strengthens the internal validity by reducing the selection and observer biases. Participant follow-up was thorough, with outcomes measured at multiple time points (intervals) post-intervention. Additionally, the study utilized the MoCA-J, which is a well-recognized and reliable tool for cognitive assessment, supporting the relevance and reliability of the cognitive function measurements.

Despite its strengths, the study has certain limitations. The sample size was relatively small and was originally calculated for a different primary outcome (patient satisfaction), which could potentially limit the statistical power regarding cognitive function outcomes. The dropout of some participants could also affect the validity of the results due to incomplete data. Although the study claims randomization, the details of the blinding procedure are not explicitly stated for all study participants. The cognitive assessments were made using only the MoCA-J, without supplementary diagnostic confirmation of MCI by a specialist, which might limit the diagnostic accuracy. The study’s crossover design may carry inherent limitations like the carryover effect, which are not thoroughly addressed.

The findings indicate that edentulous patients wearing 1-IODs had significantly increased MoCA-J scores over time, suggesting a potential benefit to cognitive function. Especially, improvements in memory and executive function scores were observed, which could have important implications for the management of cognitive health in edentulous populations. However, given the limitations, such as the potential for a learning effect on repeated MoCA-J testing and the lack of a non-crossover control group, the results should be interpreted with caution. Therefore, reported benefits to cognitive function, while promising, require validation in larger, more robustly designed studies to rule out alternative explanations and confirm the findings.

Regarding generalizability, the results show promise for the positive impact of 1-IODs on cognitive function among edentulous patients. However, the specific population studied, the unique cultural context, and healthcare settings limit the direct application of these results to broader populations. The participants were Japanese, aged 50 years or above, and had specific inclusion criteria, which may not represent the diversity of edentulous patients globally. More research is needed in varied demographic and geographic populations to establish the generalizability of these findings. Nonetheless, the study provides valuable insights that could inform further research in different contexts and contribute to a better understanding of the relationship between dental prosthetics and cognitive health.

To sum up, the RCT suggests that 1-IODs may offer cognitive advantages for edentulous elderly Japanese patients; however, more extensive long-term studies with larger cohorts are needed to validate these preliminary but promising results.

Practice points

- Single-implant overdentures may enhance cognitive health in elderly edentulous patients, with important improvements in memory and executive functions.
- Including single-implant overdentures in dental treatments for the elderly could diminish cognitive decline, extending benefits beyond dental function.
